# A Network Coding Based Routing Protocol for Underwater Sensor Networks

**DOI:** 10.3390/s120404559

**Published:** 2012-04-10

**Authors:** Huayang Wu, Min Chen, Xin Guan

**Affiliations:** 1 School of Information Science and Technology, Heilongjiang University, Harbin 150080, China; E-Mails: wuhuayang.hlju@gmail.com (H.W.); guanxin.hlju@gmail.com (X.G.); 2 School of Computer Science and Technology, Huazhong University of Science and Technology, Wuhan 430074, China

**Keywords:** underwater sensor network, time-slot based routing algorithm, balance degree, network coding, change probability

## Abstract

Due to the particularities of the underwater environment, some negative factors will seriously interfere with data transmission rates, reliability of data communication, communication range, and network throughput and energy consumption of underwater sensor networks (UWSNs). Thus, full consideration of node energy savings, while maintaining a quick, correct and effective data transmission, extending the network life cycle are essential when routing protocols for underwater sensor networks are studied. In this paper, we have proposed a novel routing algorithm for UWSNs. To increase energy consumption efficiency and extend network lifetime, we propose a time-slot based routing algorithm (TSR).We designed a probability balanced mechanism and applied it to TSR. The theory of network coding is introduced to TSBR to meet the requirement of further reducing node energy consumption and extending network lifetime. Hence, time-slot based balanced network coding (TSBNC) comes into being. We evaluated the proposed time-slot based balancing routing algorithm and compared it with other classical underwater routing protocols. The simulation results show that the proposed protocol can reduce the probability of node conflicts, shorten the process of routing construction, balance energy consumption of each node and effectively prolong the network lifetime.

## 1. Introduction

In recently years, more and more applications have appeared with the development of wireless communication network techniques [1–4]. Underwater sensor networks are an emerging and promising network technique which has attracted considerable attention. In this paper, we present a time-slot based routing algorithm (TSR) by applying a series of improvements of the flooding protocol [5]. Conflict between nodes is avoided when they start to send packets only within their own time-slots, and they don't need to reply to their parents individually in the process of establishing routing, instead they directly broadcast the routing messages. Meanwhile, to save more time and energy to quicken routing establishing process, the packet could act as the ACK to reply to their parents.

The network topology of underwater sensor networks of this paper searched is a planar centralized-tree construction. This construction has two advantages. One is easy to be extended. Tree construction can extend many branches and child branches which can be easily added into the networks. The other one is the convenience for isolating malfunctions. We can conveniently separate malfunctions from the rest of the system when nodes or routes in one branch breakdown.

**Figure 1.**
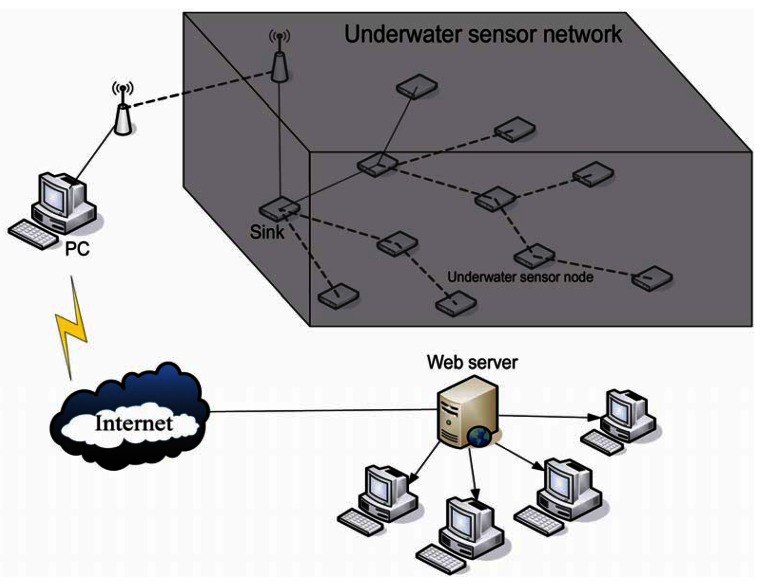
Underwater sensor network model.

As Figure 1 shows, there is one underwater sink and m underwater sensor nodes. Every node presets an unique ID (1, 2, 3, …, m). The ID of the underwater sink is 0 and the parent node of the sink is −1. All the nodes' ID will notify the underwater sink before deployment. The underwater sink connects with sensor nodes on land through wireless communication, and sensor nodes on land connect with a PC through serial ports. Thus, the PC can receive data which is collected by underwater sensor nodes through sensor nodes on land and send commands through sensor nodes on land as well as display the data on the screen and upload it to an Internet server which can be viewed and downloaded by researchers.

Due to the fact the underwater sink undertakes more missions (e.g., generating and maintaining routes, distributing channels) than underwater sensor nodes, we have to provide more high-energy battery for underwater sink.

Due to the fact TSR needs strict time synchronization, we assume each node has a strict default synchronization clock. Δ*t* is minimum time for transmission of a packet between any two nodes with no conflict. *T* ≈ *k*Δ*t*, k is revision coefficient, we set the default value is 1.5. We know all nodes' geographical location, and divide the time slot for each node based on its longitude and latitude. Before establishment of a route, the sink sends a time piece packet to a sensor node to notify which time slot they belong to. Sensor nodes only send packets in their own time slot. Through this method the control sensor nodes send packets in turns, and they can ensure the channel is unoccupied in their own time slot without overhearing the channel.

Low spreading speed and high rates of missing code are big problems for UWSNs. Therefore, it is important to quickly establish a route tree process and save the energy of nodes in a large application scenario. If we use the traditional method to establish route through polling, we need to send packets to every node and wait for ACK. As a result, every node has to wait for a long time and wastes too much energy. Therefore, it is very important to use time appropriately and decrease the depletion of energy. In such cases, only by ensuring every node has an equal opportunity to send packets can decrease the waiting time. This paper presents TSR based on a flooding protocol to save waiting time. Nodes can send packets in their own time slot no matter if the last node is done sending or if it has received a waiting packet. The nodes broadcast the packets which they are received. Though this process we can decrease the amounts of packets and increase the rate of route construction. We will show the details in Section 2.

To balance the network load and increase the network lifetime, this paper presents an equal probability method. In the route tree establishment process, the network adjusts the number of each route tree's sub-node according to the equal probability method to equilibrate the probability of each route tree. Therefore, the network can use each node equally and avoid some nodes exhausting their energy prematurely. The network lifetime would be extended by using this method.

Furthermore, we introduce the network coding theory and combine it into the TSBR algorithm. In the traditional network communication, the forward method is store-and-forward, whereby intermediate nodes only forward packets and do not process anything. Based on this, the authors of [6] proposed a new forwarding method and named it network coding theory. Network coding is considered as a generalization of conventional store-and-forward routing techniques and it was originally proposed in order to achieve multicast data delivery at the maximum data transfer rate in single-source multicast networks. This feature had a great impact on the research field of information theory and research on network coding was first activated in the information theory community.

Network coding is a new forward method that takes route and coding into consideration. In network coding theory, an intermediate node can process the received packets with linear or non-linear transforms [7] and then forward the processed packets.

The basic idea of network coding is that each node processes the data which are received from each channel with a linear non-linear transform and forward it to the next hop. Based on the maximal flow-minimal cut theorem in graph theory [8], sender and receiver of data communication cannot communicate with the rate greater than maximal flow (or minimal cut). If we use traditional multi-broadcast-route method, it cannot achieve the maximal flow. Ahlswede and co-workers used a butterfly network as the instance of their research [9], and figured out that the use of network coding can achieve the maximum flow rate and improve communication efficiency.

Currently, network coding theory is widely used in wireless networks, P2P network systems, distributed file storage, and network security. However, because of the particular situation of under-water networks, network coding theory needs a broader systemic study [10].

## 2. Routing Protocol Design

In this section we will introduce the routing protocol of underwater sensor networks. Firstly, we present TSR then extend to TSBR by adding a probability balanced mechanism to TSR. Furthermore, we add network coding theory into TSBR and have thus obtained a more efficient routing protocol.

### 2.1. TSR Design

Nodes in UWSNs have to send route packets to establish a route tree. The first step is to check the format of the route packet. Nodes do not have to reply to their parent node, instead of broadcasting the route packet directly. The route packet also can be seen as the affirmance of the parent node, as well as taking route messages of all nodes to the sink. Therefore, we divide packets into broadcast-route packets and feedback-route packets during the process of route establishment.

**Figure 2.**
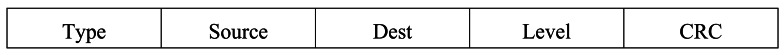
Format of a broadcast-route packet.

Broadcast-route packets are mainly used to find the next hop directly and can be sent to a parent node as ACK. They contain five fields in the format shown in Figure 2.

**Figure 3.**
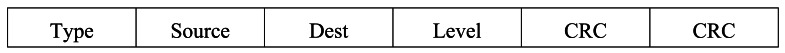
Format of feedback-route packets.

Feedback-route packets are mainly used by lower layers to report to the parent node the construction of a route and be sent to a sink. A sink stores route packets in its buffer to check each state of each node. Feedback-route packets contain seven numeric fields, in the format shown in Figure 3.

The meanings of the route format fields are shown as the following:

Type: type of packet, 0 denotes broadcast packets, 1 denotes feedback packets
Source: ID of node which sends a packet
Birthtree: ID of node which creates a packet, and starts to send it back to a sink
BTParent: ID of Birthtree's father node
Dest: Packet destination
Level: layer the node which creates a packet belongs to
CRC: circulating redundancy check message

**Figure 4.**
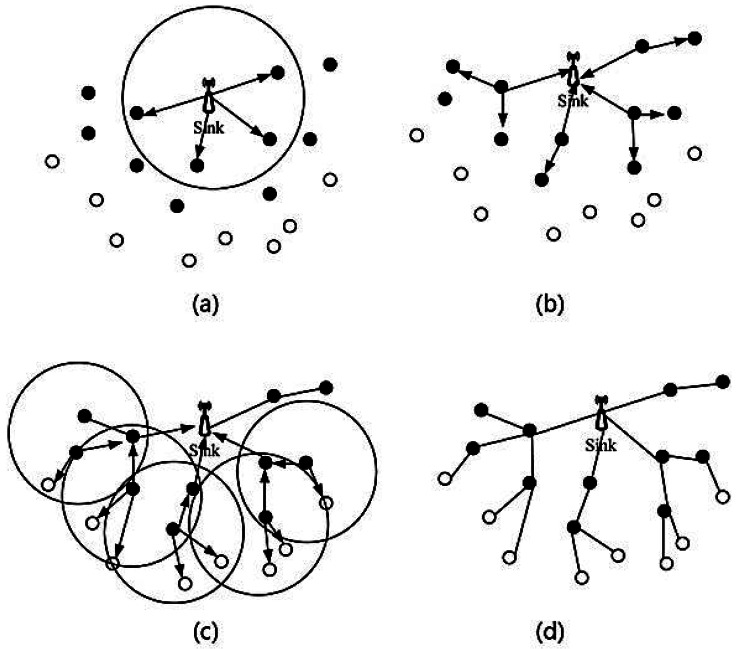
(**a**) Sink sends broadcast-route packet. (**b**) First layer nodes send broadcast-route packets. (**c**) The second layer nodes send broadcast-route packets. (**d**) Established route tree.

The basic TSR establishment method is: sink sends the broadcast packet during the first period in its own time slot. Each node which received the broadcast packet sends back a feedback packet in a particular period in a given time slot. If the sink received the feedback from nodes, the sink will register these nodes, which are the child-nodes of the sink, as first layer child-nodes. When a node first receives a packet which is not come from the sink, it would determine the current time slot and whether it had missed its own time slot. If not, it will broadcast the packet in its own time slot and if yes, it would wait for another own time slot. If node *x* received a packet from other node *y*, it registers *y* as its sub-layer. Then, it sends the packet to parent-node *z*. Node *z* also sends a packet to its parent-node *a*, until the parent-node is the sink. This process will continue until all nodes are registered. The interior communication process through a route tree would decrease conflict probability efficiently and decrease the establishment time of a route tree. The process of establishing a route tree is shown in Figure 4.

To explain this more clearly, we divide the process of establishing a route tree into a sink route establishment process and a normal route tree establishment process. In the route establishment process a sink sends a broadcast route packet, and then just waits for packets whose destination is the sink. The other underwater nodes have to determine the received packet's type when they first receive it.

The TSR design process can be listed as follows:

1. Sink send packet <*0, 0*, −*1, 0, CRC*> during the first period in a given time slot *a_1_T*.
2. Any node *x*, whose time slot is denoted by *a_2_*, broadcasts its own packet <0, x, 0, 1, CRC> in a particular time slot if it receives a packet from a sink.
3. Any node y (time slot is *a_4_*), in the case it did not receive a packet from a sink, but from another node *z* for the first time (packet is <0, *z, z_Patent_, n, CRC*>), and if *y* > *z, y* will broadcast packet <0, *y, z, n*+*1, CRC*> in its own time slot in the next period.
4. If node *z* received a packet from node *y*, then it registers *y* as its sub-node (in the *n*+*1* layer)
5. Any non-sink node *x* (time slot is *a_5_*), after receiving a packet<*0, y, x, a, CRC* > from another node *y*, will send back a packet <*1, x, y, x, x_Parent_*, *a, CRC*> in its own time slot in the next period if *x*>*y*. Node x will integrate all packets (such as from x and y) when received in the same period into one packet<*1, x, y, z, x, x_Parent_, a, CRC*>.
6. Any non-sink node x_1_ (time slot is *a_7_*), when it receives a feedback packet<*1, x, y, x, x_Parent_, a, CRC*> sent to it from another node (time slot is *a_8_*), it will send back a packet <*1, x_1_, y, x, x_1Parent_, a, Info*> to its father-node *x_1Parent_* in its own time slot if *x_1_* > *x*. Otherwise it sends it in its own time slot *a_7_T*. If *x_1_* received packets from more than one node, it will integrate the messages of all nodes into one packet and determine whether the packet size is more than 1 K bits. If yes, it then sends it in its own time slot. Otherwise, it divides the packet into several packets and orders them based on the time of reception and it will send the packet in the next *a_7_T* time slot.
7. Repeat the above process, until all nodes have been registered.

In TSR, each underwater node has to maintain two tables: a state table and a table of destinations. The state table contains residual energy, total data size of sent packets, throughout capacity, error rates, average delay and repeat send times. The table of destinations contains the addresses of destinations, next hop addresses, and time messages created by the route. The sink has to know the message status for all nodes and the node stable of all source nodes.

Since the network power is supplied by batteries and it is difficult to replace batteries, we must pay more attention to reducing the consumption of energy to avoid the failure of a whole underwater network because of a few nodes whose energy is exhausted. A node seeks a route table firstly when it wants to send data which it has collected by itself or to forward data coming from other nodes to choose a route which has less hop time and more energy. Every node would update the current route table and node status table after a data sending action. This management model can ensure packets will be forwarded along an optimal route to the sink.

Based on the above algorithm, underwater sensor nodes can communicate with each other through the established route. However, affected by inescapable factors, such as channel conflicts, noise jamming, overhearing and encumbrance, nodes may disconnect and underwater networks may break down. Because of their limited energy, underwater networks may also break down when some node has exhausted its power supply. In such case, we have to maintain networks in a timely fashion.

Breakdown situations can be divided to two categories: one is when some nodes have exhausted their energy; the other one is that the packets are missed in the forwarding process. We present two kinds of methods to resolve the above problems.

#### (1) Resolution method when a node has died

In the packet forwarding process, some underwater sensor nodes may exhaust their energy because of oversized communication. Thus, the underwater sensor network breaks down as a result. We employ the following strategy to resolve this situation: in the sending process, if node A has found the next hop node died because of energy exhaustion, it will broadcast a route maintaining packet in its own time slot. A node which receives the route maintaining packet will reply to node *A* with one packet containing its residual energy and sub-node number. Node *A* would compare all the reply packets and select the node that has least sub-nodes as next hop. If the sub-node number is equivalent, then it selects the node which has most energy as the next hop.

#### (2) Resolution method for missing packets

We propose a repeated sending method to resolve missing packets. Each underwater node has a buffer to store packets. A node will send a packet again if it didn't receive the feedback packet when the clock times out. If it has repeatedly sent one more than three times, then it confirms the next hop died, and the method of Section 2.4.1 would be processed.

### 2.2. TSBR Design

To balance the network burden and increase lifetime, we propose an equal probability method. In the route tree establishment process, the network adjusts the number of each route tree's sub-nodes according to the equal probability method to equilibrate the probability for each route tree. Therefore, the network can consume the energy of each node equally and avoid some nodes exhausting their energy prematurely. Network lifetime would be prolonged through this protocol. In the following content, we will present some concepts about equilibrium:

#### Definition 1: equilibrium

If route tree *T* is in equilibrium, then *T* must satisfy: *T* is null or there is no more than *1* different values of neighbor nodes in the same layer.

#### Definition 2: equilibrium factor

If one node's low layer has several nodes, *i*′ is the node who has the least sub-layers. Its sub-node number is $k_{i\prime }$, equilibrium factor is *x_i_*_′_ = *1*. The sub-node number of another node *i* is *k_i_*. The different value between each node and the node has least sub-node is *α* = *k_i_* + *k_i_*_′_. The equilibrium factor is:

(1) $$\begin{array}{cc} x_{i}=1/(\alpha +1) & 0<x_{i}\leq 1 \end{array}$$

This paper use equilibrium degree to measure whether a route is equal. The more the equilibrium degree, the more equal the route, the more equal the consumption of energy and the more prolonged the network lifetime. Equilibrium degree is defined in Definition 3.

#### Definition 3:

Equilibrium degree is a linear set of its sub-nodes, formatted as follows:

(2) $$\gamma =\{\begin{array}{cc} 1 & i=0,1\\ \frac{1}{\alpha }\sum_{i=1}^{n}x_{i}\gamma_{i} & i=2,3,\ldots \end{array}$$

where *γ* is equilibrium degree, *n* is sub-node number of the node, *γ_i_* is the *i_th_* sub-node of the node, *x_i_* is the equilibrium factor.

**Figure 5.**
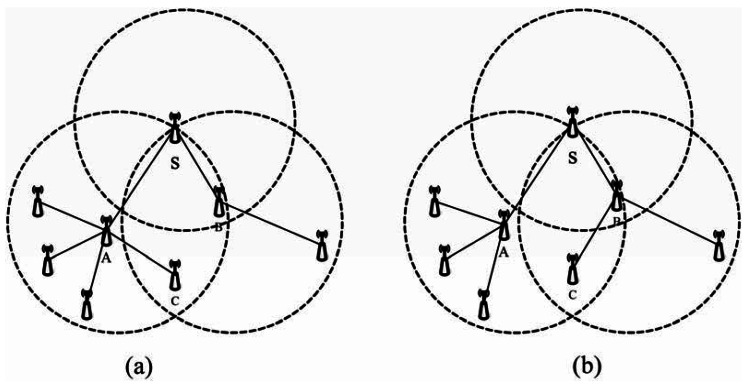
Balancing process. (**a**) No balancing method is applied. (**b**) Balancing method is applied.

The following sections show the equal route tree algorithm is just an approximate probability equilibrium which cannot achieve non-conditional equilibrium. The main idea of this algorithm is to add a numeric *SubNodeNum* field into a broadcast-route packet and feedback-route packet to give the sub-node number of the next layer whose default value is 0. The *SubNodeNum* will be plus 1 if its node adds a new sub-node. Node *A* has more sub-nodes than its neighbor node *B* and the different value is *a*. Meanwhile, node *C* is in the communication range of *A* as well as *B* as is shown in Figure 5, where *C* is registered as a sub-node of *A* after receiving a broadcast-route packet which come from A, then receiving a broadcast-route packet from *B*. If *C* knows *A* has more sub-nodes than *B* and the value difference is more than 1, it will quit from *A* and then register as a sub-node of B. Therefore, the difference between the sub-node numbers of *A* and *B* has decreased. In such way, we obtain an approximately equal route tree.

In Figure 5, after a route tree has been established, *A* has four sub-nodes and *B* has one sub-node. *A* will consume more energy than *B* in the same situation. In such a case, *A* would die earlier than *B*. We can conclude from the equilibrium definition that the equilibrium factor of node *S* in Figure 5(a) is 1/4 and the equilibrium degree is 5/9. Therefore, the network is unequal. After using TSBR, the sub-node number of *A* decreases to three, and that of *B* increases to two. The equilibrium of node *S* in Figure 5(b) is 1/2 and the equilibrium degree is 5/6. Due to this equilibrium is the most important factor to measure if a network is equal. We can confirm the network would become more equal by using TSBR and thus more suitable for underwater sensor networks.

According the basic idea of TSBR, we deduce the probability of a node *A* changing to become *B*'s sub-node. Assume *A* has more sub-nodes than *B*, and the sub-node number of *A* in the communication range of *A* and B is k. The sub-node number of *A* out of the common communication range is *k*_1_; *k_2_* is the sub-nodes number of *B*.

The different value of sub-node number between A and B is:

(3) $$\begin{array}{cc} \alpha =k_{1}+k-k_{2} & \alpha >1 \end{array}$$

The different value of sub-node number between A and B exceeds common communication range is:

(4) $$\beta =k_{1}-k_{2}$$

We calculate the probability change according to the follow two situations:

$$\begin{array}{cc} •\text{if}\;k=0 & \;P=0\\ •\text{if}\;k>0 & \{\begin{array}{cc} \text{if}\;\beta <0 & P\in (0,1/2)\\ \text{if}\;\beta =0 & P=1/2\\ \text{if}\;0<\beta <k & P\in (1/2,1)\\ \text{if}\;\beta \geq k & P=1 \end{array} \end{array}$$

We analyze the situations of *â* < *0* and *0* < *â* < *k* when *k* > *0*. Based on the definition of equilibrium, we realize equilibrium means to make the sub-node number of each node as equal as possible. We can achieve the target by giving [*á/2*] nodes to *B* when node number k in the common communication range is greater than 0, and *â* < *0, 0* < *β* < *k*, the change probability of a sub-node in the common communication range is [á/2]/k.

Therefore, we can conclude the change probability of *k* nodes based on the above analyses and large amount data fitting as follows.

(5) $$p=\{\begin{array}{cc} 0 & k=0\\ 1/2 & \;k\neq 0,\beta =0\\ 1 & k\neq 0,\beta <k\\ \left[\left|\frac{\alpha }{2k}\right|\right] & \text{others} \end{array}$$

In the process of route establishment, we can use Equations (3–5) to conclude the change probability of a node in common communication range to dynamically adjust the sub-node number of each node.

### 2.3. TSBNC Design

**Figure 6.**
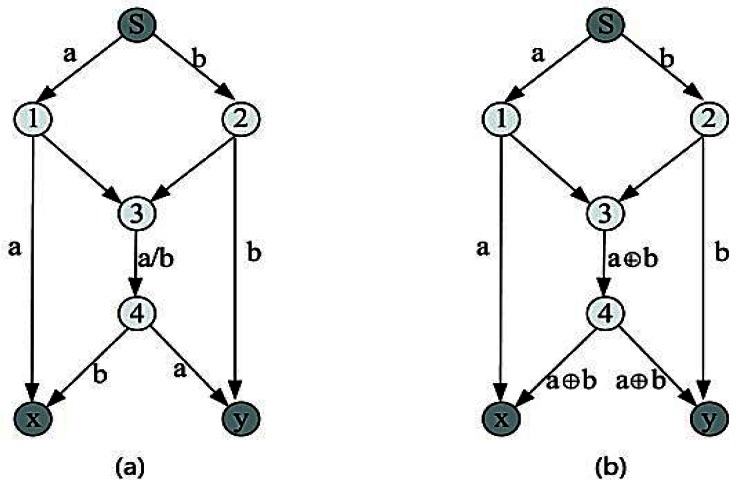
Comparison between coding and routing: (**a**) Store-and-forward method. (**b**) Coding method.

Compared with a traditional network, our network mainly uses the multicast transmission method [11]. This has immense advantages in increasing throughput rate, improving the stability of communication, a simple management strategy and so on [12]. The essence of network coding is to use the computation of nodes to improve the bandwidth efficiency. Its work principle is shown in Figure 6(b). *S* is the source node, *x* and *y* are destination nodes. Each channel has 1 bit/s bandwidth. If we forward 1 bit data (a, b) from *S* to *x* and *y* using the traditional method, we have route 3→4, as can be seen in Figure 6(a). Since *a* and *b* cannot go through the route 3→4 in the same time, we only can use the store-and-forward method; its maximum data flow is 1.5 bit/s. If we use network coding method, node 3 codes *a* and *b* with XOR operation into a new packet and forwards it to *x* and *y* through node 4. In such a case, *x* can obtain the packet through a decoding method as well as *y*. The maximum flow rate is 2 bit/s and the bandwidth utilization ratio is thus increased 33%.

After the establishment of the routes, each node has at least one route to the sink. Nodes will send the collected data to the sink following this route. To further save energy and bandwidth, we employ network coding in the forward process. Intermediate nodes code several received packets into one coding packet, and forward it to the next hop. Thus, we can extend the network lifetime.

Two nodes can forward each other's packets when they are in the same communication. Assume *A* and *B* are two nodes that are connected. Packet *E* is the linear combination of several source packets (*X_1_, X_2_*, …, *X_k_*). $E=\sum_{i|1}^{K}\alpha_{i}X_{i\prime }$, where *á* is the coding coefficient.

1. Node *A* codes all the packets which are in its buffer (*y_1_, y_2_*, …, *y_m_*) into one coding packet *E_a_*.
  (6) $$E_{a}=\sum_{i=1}^{m}\beta_{i}y_{i}$$
  where *â_i_* is chosen from the Galois field randomly.
2. Node *A* forwards the packet *E_a_* and coding coefficient to *B*.
3. Node *B* will put E_a_ into a buffer if it has enough space when it receives *E_a_*, otherwise it codes E_a_ with packets in the buffer with the following equation:
  $$E_{i}\prime =E_{i}\prime +\alpha E_{\alpha }$$
  where *E*′*_i_* is the *i_th_* packet, *a* is chosen from the Galois field.
4. The process will continue until the sink has received the coded packets from other nodes.
5. The sink will decode the received k packets. Realized from coding coefficient and coded packets, every packet represents a linear equation that contains *k* sources as the unknown element. This decoding matrix represents the coefficient matrix for the linear system. If *K* is the order of the matrix, the sink will continue to receive packets from other nodes.

In the process of forwarding packets, intermediate nodes will code all the packets in their buffer and forward them to the next hop. Thus, we can decrease the number of packets and further decrease the consumption of energy and increase the bandwidth utility ratio.

## 3. Simulation Results and Discussion

In this paper, we use NS2 (Network Simulator version 2) as the simulator. NS2 [13] is an object-oriented simulation tool and it is a discrete event simulator with a virtual clock, that drives all simulations by discrete events.

In underwater sensor networks, since the environment is totally different from the land scenario, the channel quality is also worse than in traditional wireless sensor networks. The difference compared with traditional WSNs is that acoustic communications become the physical layer technology in underwater networks and the available bandwidth is severely limited when applying underwater acoustic channels. The underwater channel is severely impaired due to multi-path interference and fading. Propagation delay is another challenging issue. The delay time in underwater environments is five orders of magnitude higher than in radio frequency (RF) terrestrial channels and it varies. This fact may lead to unsuccessfully transmission between nodes due to the uncertain delay.

When we perform a simulation to evaluate the performance of the proposed routing protocol, we also consider the channel quality issue. In such a case, we implement our routing protocol on the NS-2 simulation platform. Since the NS-2 platform has fully considered the underwater environment, the simulation results are very close to the real underwater sensor networks. In order to understand the impact of channel contention and the interaction of multiple flows in the networks, the underwater acoustic channel model had been implemented in NS-2. The NS-2 implementation includes the propagation time model, the bandwidth-distance relationship and the attenuation and signal-to-noise ratio for three-dimensional underwater sensor networks.

The existing well designed routing protocols for mobile wireless sensor network are hard to implement due to the unique characteristics of underwater sensor networks. First of all, radio does not work well in water because of its rapid attenuation. Thus acoustic communications are usually adopted in underwater environments. Acoustic channels often feature low bandwidths and long propagation delays. Thus a routing protocol with long end-to-end delays or high bandwidth requirements is not a good choice. Secondly, most nodes in a UWSN can move passively with water currents (except that some gateway nodes are fixed on the water surface or anchored to the bottom), resulting in a highly dynamic network topology. To handle dynamic networks, existing routing protocols for land-based sensor networks need to update routing information periodically, which introduces significant communication overhead. Thirdly, like land-based sensor nodes, underwater sensor nodes are usually powered by batteries, which are even harder to recharge or replace in harsh underwater environments. Thus, energy efficiency is another important concern for UWSN routing.

There are three categories of dynamic routing protocols. Proactive routing protocols maintain the route periodically in order to create a fresh enough route for data transmission. However, periodically route maintenance decreases available limited bandwidth. Reactive routing protocols are on demand. They build routes between nodes only as desired by source nodes. Some research has tried to strike a balance between proactive and reactive routing as a hybrid mode. Conventional proactive routing protocols rely on systematic flooding for route discovery and maintenance, potentially causing excessive energy consumption and collisions. Geographic routing protocols need help from a globe positioning system (GPS). These protocols are not designed for underwater sensor networks. In an underwater sensor network scenario, general 3D geographic routing is preferable as it is stateless. However, geographic routing requires online mode, distributed localization of mobile sensors which is expensive and takes a long time to converge.

In consideration of the above reasons, we do not compare our proposed routing protocols with the existing well designed routing protocols and only compared the performance of our protocols with the classical routing protocols which have been proved to work well in underwater scenarios. The simulation parameters are listed in Table 1.

### 3.1. Network Lifetime Simulation and Results

We define the network lifetime as when 10% of nodes have exhausted their energy. Along with the increasing number of nodes, network lifetime decreases mainly because the increased number of sub-nodes make the source node consume more energy.

**Figure 7.**
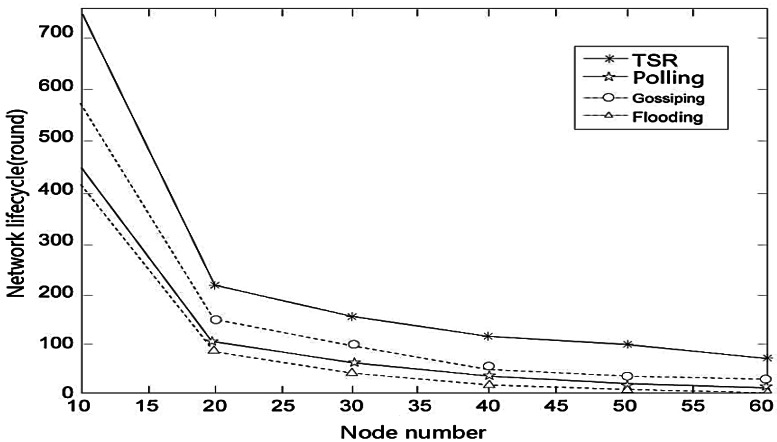
The network lifecycle under different node number.

We can see in the Figure 7 the lifetime of TSR, gossiping protocol, polling algorithm and flooding protocol decrease rapidly when the node number is increased to 20 from 10. We can see that TSR has the longest lifetime compared with the other three protocols.

### 3.2. Simulation Results for Establish Time of Route

**Figure 8.**
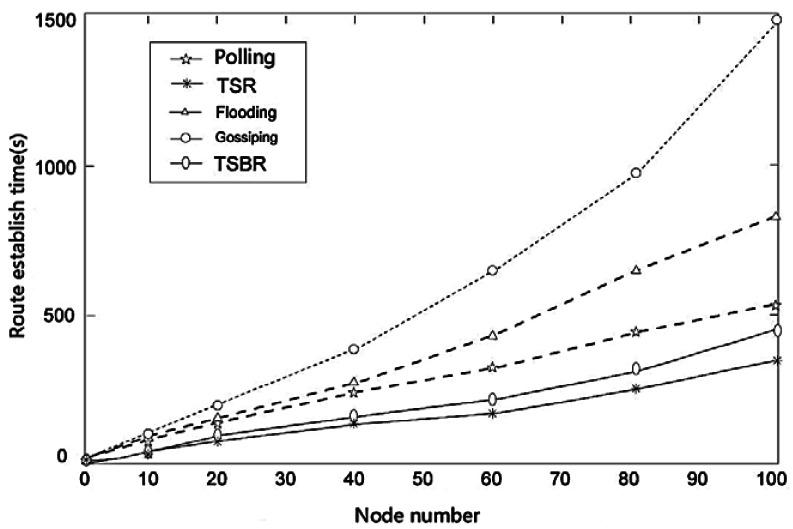
Route establishment time under different node numbers.

We simulate the network establishment time of TSR, TSBR, gossiping protocol, polling algorithm and flooding protocol. We can see from the results in Figure 8, TSBR takes more time to establish a network than TSR, but still less than the other protocols.

### 3.3. Equilibrium Simulation and Results

**Figure 9.**
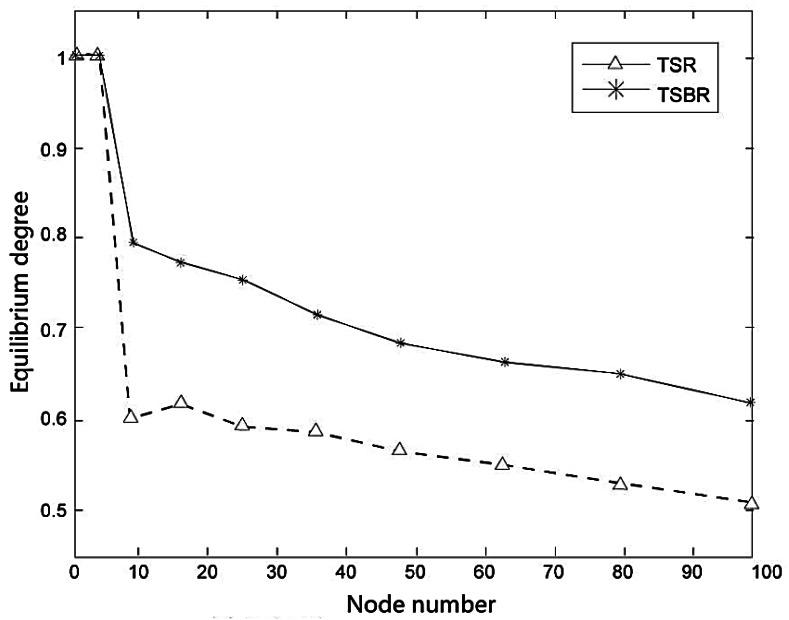
Equilibrium degree for different node numbers.

The condition of route equilibrium will have a significant effect on network performance. Figure 9 shows the equilibrium condition for different node numbers.

We can see from the Figure 9 that the route equilibrium of both TSR and TSBR decrease with the increasing number of nodes, but TSBR performs better than TSR.

### 3.4. Energy Consumption Comparison

**Figure 10.**
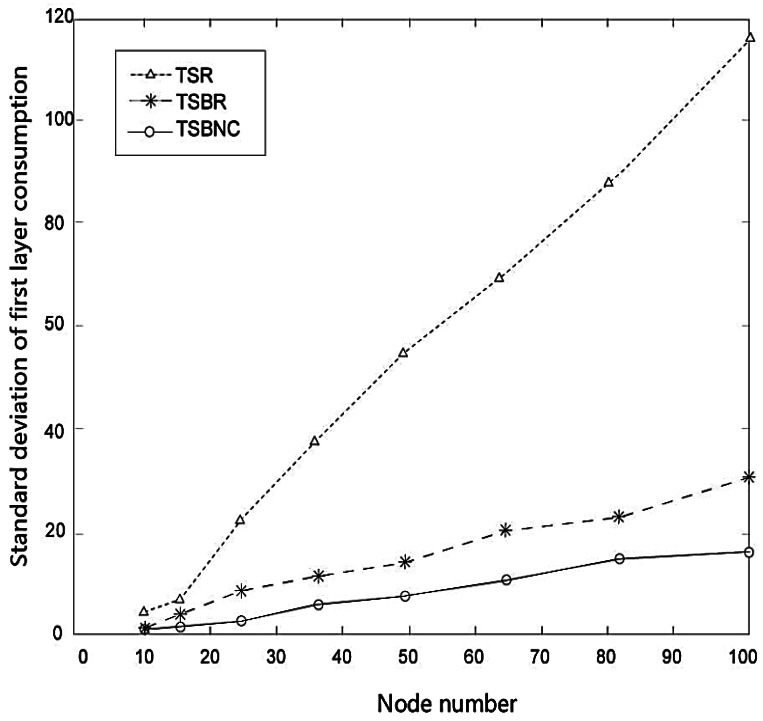
Standard deviation of first layer consumption.

We also compare TSR and TSBR on their energy consumption performance. Figure 10 shows the standard deviation of the first layer node consumption change with increased node number. The results show that all three protocols consume more energy if node number is increased, but we can see clearly that the extent of the increase of TSBR is much lesser than for TSR. Therefore, we can confirm TSBR improves the equilibrium. The advantages perform better with increased number of nodes. Furthermore, TSBNC further decreases the consumption based on TSBR.

**Figure 11.**
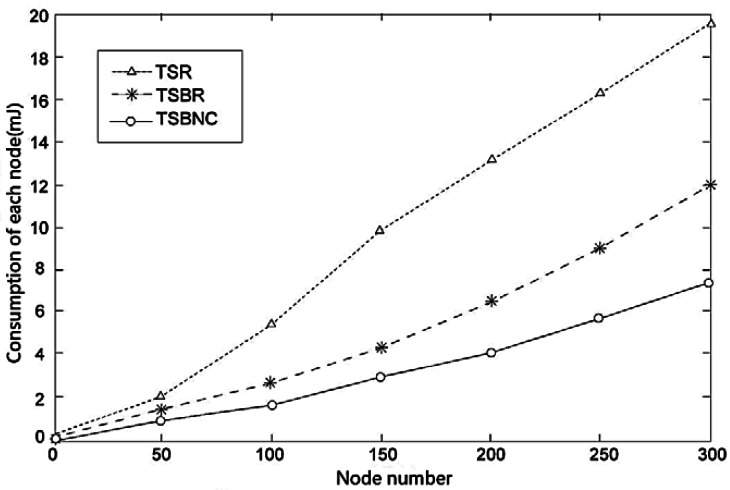
Consumption of each node for different node numbers.

We also confirm the advantages of TSBR and TSBNC in Figure 11, which shows how the consumption of sending one packet changes with increased node number. The increase of TSBNC is the slowest among the three protocols. TSBR's performance is worse and TSR's performance is the worst among the three protocols. The results prove TSBNC has excellent advantages when equalizing the node consumption.

### 3.5. Algorithm Verification

This paper uses the MAC protocol based on TDMA, and divides each period to nine time slots, and in each time slot, only one node can send or receive a data frame. The authors of [14] presented a model which can be used to quantify the consumption of energy of underwater sensor networks. The following content will describe the model in detail: assume the minimum power of a node receiving packets is *P_0_*. If a node sent *i* bits packet to the another node at a distance *l*, the consumption is:

(7) $$E_{s}(i,l)=iP_{0}l^{k}\alpha^{l}$$

The consumption of the other node is:

(8) $$E_{R}(i)=iP_{r}$$

where *i* is the bit rate of the sent packet, *l* is the transfer distance, P_r_ is a constant parameter which is decided by the receiving device, *k* is the energy coefficient (for a cylindrical shape k is 1–1.5 and k is 2 for roundness). *á* = *10á^á^*^(^*^f^*^)^*^/10^, á*(*f*) is absorption coefficient measured in dB/km relevant to the frequency. Based on the Thorp function we can calculate the absorption coefficient of the frequency range of interest:

(9) $$\alpha (f)=0.11\frac{f^{2}}{1+f^{2}}+44\frac{f^{2}}{4100+f^{2}}+2.75\times 10^{-4}f^{2}+0.003$$

To simulate the underwater environment, we have to realize the parameters that will affect the simulation first, then enactment these parameters such as bit error rate, network delay, noise, conflict and overhearing.

Bit error rate [15] is the indication of the measure exactness of data in the scheduled time:

(10) $$\text{bit error}=\frac{\text{bit error in transmission}}{\text{total bit in transmission}}\times 100\%$$

**Figure 12.**
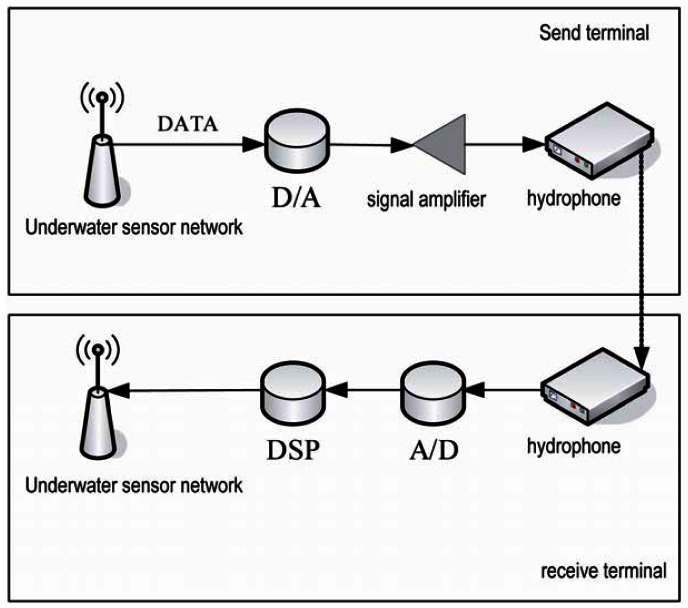
Communication process of an underwater sensor node.

In the application to underwater sensor networks, nodes communicate with each other by sound waves. At the sending terminal, nodes need to transform digital signals into analog signals and amplify them with an amplifier before sending them through a hydrophone. The receiving terminal receives the analog signals and then transforms them into digital signals. The process is shown in Figure 12. The transformations and the complex underwater environment will increase the bit error rate [16].

There are two main methods for measuring bit error rate: statistics and actual measurement [17]. This paper used measurement to obtain the underwater bit error rate. Through a large amount of experiments in the actual environment and calculations, we set the bit error rate as follows:

(11) BER=2.257le−4

Network delay [18] is the time spent to send a packet from a terminal to the other terminals that include sending delay, transmission delay and processing delay.

total delay=send delay+transmission delay+process delay

Sending delay is the time cost of the *D/A* transform [19], power amplification and underwater transformation. Transmission delay is the time cost caused by the transmission of sound waves underwater. Processing delay is the time cost caused by wave filtering, *A/D* transform, and digital signal processing. We assume *T1* is the sum of send delay and processing delay, *T2* is the transmission delay. Through a large amount of experiments in the actual environment and calculations we set the delay as follows:

(12) $$\begin{array}{l} T1=118ms\\ T2=1520\times L \end{array}$$

where *L* is the distance between any two nodes.

Noise [20] is caused by waves, currents, ships and marine organisms. We evaluate it in an actual environment under different weather and sea conditions. We adopt two approximate noise probabilities: the higher noise is 7.6‰ and the lower noise is 3‰.

If two nodes use the same channel in the same time slot, they will interfere with each other breaking the packet and cause conflict. Because conflict cannot happen between PCs, we have to simulate underwater conflict to test the reliability of protocols. We conclude the conflict situation as follows:

1. For the packet which has a destination, we only check whether the neighbor nodes are also forwarding the packet to the same destination.
2. To broadcast a packet, we check whether other nodes are sending in the same slot.

Due to the fact underwater sensor networks share communication channels, nodes perhaps receive some packets that they should not receive. This situation is known as overhearing and will cause more consumption of energy [21]. Overhearing exists in the practical environment, so we have to consider the overhearing issue in our simulation to approach the practical underwater transmission environment.

We use network topology to imitate overhearing [22]. We import the network topology into a neighbor table, and a node send packets to all neighbors when the packet needs to be sent to the next hop. So, the neighbor will experience overhearing. The simulation has the following steps:

1. Input node number, condition, and node's IP in the PC.
2. Through the distance between every two nodes calculate each node's neighbor.
3. Send start order, and nodes begin communication.
4. The simulation program calculates send delay from the packets received from nodes by socket, puts packets into a buffer and send them in its own time slot.
5. The node adds bit error and noise according to the bit error rate to check whether conflict will occur. If yes, it records conflict messages and processes the packet with some operation.
6. Send packet to all neighbors.

We perform the laboratory simulation with nine nodes. We divided nine time pieces for nodes according to their location that was preset before simulation. The results showed the consumption of the nodes and the total data is in equilibrium, the average delay and bit error rate are low.

## 4. Conclusions

Underwater sensor networks are a newly arisen technique compared with traditional wireless network applications, and it is expected that they will be widely used in the near future. This paper studied in depth the routing problems of underwater sensor networks and presented novel routing protocols to solve these problems. Firstly, we presented TSR by having done a series of improvements on the flooding protocol. Secondly, we presented TSBR by adding a probability balanced mechanism to TSR. After that, we proposed TSBNC by introducing network coding theory into the TSBR algorithm. Finally, we evaluated the performance of TSBNC. The results showed that our proposed protocols can reduce the probability of node conflicts, shorten the process of routing construction, balance energy consumption of each node and effectively prolong the network lifetime. Underwater sensor networks are complex scenario which needs to be studied insistently and our future work will further focus on mobile underwater sensor networks to build a practical environment and to test our protocols.

## Figures and Tables

**Table 1.** Simulation parameters.

Routing protocols	TSR, TSBR, TSBNC
MAC protocol	Based on TDMA
Area	1,000 m × 1,000 m
Forward model	Underwater communication
Node distribute	Random
Node number	100
Communication range	10 m
Delivery probability	90%
